# The complete chloroplast genome sequencing analysis revealed an unusual IRs reduction in three species of subfamily Zygophylloideae

**DOI:** 10.1371/journal.pone.0263253

**Published:** 2022-02-02

**Authors:** Xiaoyang Wang, Tashi Dorjee, Yiru Chen, Fei Gao, Yijun Zhou

**Affiliations:** 1 Key Laboratory of Ecology and Environment in Minority Areas (Minzu University of China), National Ethnic Affairs Commission, Beijing, China; 2 College of Life and Environmental Sciences, Minzu University of China, Beijing, China; National Cheng Kung University, TAIWAN

## Abstract

*Tetraena mongolica*, *Zygophyllum xanthoxylon*, and *Z*. *fabago* are three typical dryland plants with important ecological values in subfamily Zygophylloideae of Zygophyllaceae. Studies on the chloroplast genomes of them are favorable for understanding the diversity and phylogeny of Zygophyllaceae. Here, we sequenced and assembled the whole chloroplast genomes of *T*. *mongolica*, *Z*. *xanthoxylon*, and *Z*. *fabago*, and performed comparative genomic and phylogenetic analysis. The total size, structure, gene content and orders of these three chloroplast genomes were similar, and the three chloroplast genomes exhibited a typical quadripartite structure with a large single-copy region (LSC; 79,696–80,291 bp), a small single-copy region (SSC; 16,462–17,162 bp), and two inverted repeats (IRs; 4,288–4,413 bp). A total of 107 unique genes were identified from the three chloroplast genomes, including 70 protein-coding genes, 33 tRNAs, and 4 rRNAs. Compared with other angiosperms, the three chloroplast genomes were significantly reduced in overall length due to an unusual 16–24 kb shrinkage of IR regions and loss of the 11 genes which encoded subunits of NADH dehydrogenase. Genome-wide comparisons revealed similarities and variations between the three species and others. Phylogenetic analysis based on the three chloroplast genomes supported the opinion that Zygophyllaceae belonged to Zygophyllales in Fabids, and *Z*. *xanthoxylon* and *Z*. *fabago* belonged to *Zygophyllum*. The genome-wide comparisons revealed the similarity and variations between the chloroplast genomes of the three Zygophylloideae species and other plant species. This study provides a valuable molecular biology evidence for further studies of phylogenetic status of Zygophyllaceae.

## Introduction

*Tetraena mongolica*, *Z*. *xanthoxylon*, and *Z*. *fabago* are three typical dryland plants belonging to the subfamily Zygophylloideae of Zygophyllaceae [1–3]. Zygophyllaceae are a family of about 350 species in 27 genera, and plants in Zygophyllaceae are mainly distributed in tropical, subtropical and temperate regions in Asia, Africa, Europe, America, and Australia. In China, there are 5 subfamilies, 6 genera, 31 species, 2 subspecies, and 4 varieties of Zygophyllaceae plants. Zygophyllaceae plants are usually herbs, shrubs, or subshrub, and most of them are tolerant to drought and salt stress, and can grow in barren soil in the arid and semi-arid regions, that represents high ecological importance of the species.

There are different points of view on the taxonomic status of Zygophyllaceae. According to Cronquist system [3], Zygophyllaceae was classified into Sapindales based on its morphological characteristic. However, Zygophyllaceae was classified in the order of Geraniales in *Flora Reipublicae Popularis Sinicae* [4] and *Flora of China* [5], based on the morphological feature. The angiosperm taxonomy published by the angiosperm phylogenetic group (APG) from 1998 is making efforts to build a consensus view of the taxonomy of flowering plants based on DNA sequence data [6]. In APG IV system published in 2016 [7], Zygophyllaceae were included in Zygophyllales, and Zygophyllales was classified in the legumes of Rosids in the core eudicotyledons, as a base group of this branch. In addition, according to *Flora Reipublicae Popularis Sinicae*, there are six genera in Zygophyllaceae, including *Tetrahedral*, *Sarcozygium*, *Zygophyllum*, *Nitraria*, *Peganum*, and *Tribulus*, but in *Flora of China*, there are only three genera included in Zygophyllaceae, *Tetrahedral*, *Zygophyllum*, and *Tribulus*. *Sarcozygium* was classified into *Zygophyllum*, and *Nitraria* and *Peganum* are classified as two new families, Nitrariaceae and Peganaceae. More evidence is needed to clarify these taxonomic issues.

The chloroplast genome is a suitable tool for studying evolution and phylogenetics of plants because of its highly conserved sequence and structure [8]. As one of the two semi-autonomous organelles in plant cells [9], chloroplast is the main site of photosynthesis [10, 11]. The chloroplast genome of most angiosperms is inherited from the maternal line, while that of gymnosperms is mainly inherited from the paternal line [12]. In general, chloroplast genome exhibits a typical quadripartite structure, ranging in size from 120kb to 200kb, including a double-stranded closed loop with a long single-copy sequence (LSC, 80 kb-90 kb), a short single-copy sequence (SSC, 16 kb-27 kb), and two reverse repeat sizes (IRs, 20 kb-28 kb) with roughly equal length [9, 13]. The nucleotide sequence of chloroplast DNA provides a large amount of information, including not only related information on protein-encoding and non-coding genes, but also data to infer gene rearrangement and evolutionary relationships [14, 15]. The chloroplast genome has become an indispensable molecular resource for species identification, molecular barcode, population genetics and phylogenetic research [16–18], also the comparative analysis based on chloroplast genomes reveals gene rearrangement events and evolutionary histories.

Previous studies have reported the chloroplast genomes of *T*. *mongolica*, *Z*. *xanthoxylon*, and *Z*. *fabago*. However, further studies are needed to deeply explore the structure and phylogenetic status of the three species [19, 20]. In the present study, the complete chloroplast genomes of *T*. *mongolica*, *Z*. *xanthoxylon*, and *Z*. *fabago* were sequenced by using Illumina sequencing platform, and then assembled and annotated. Comparative genomics tools were used to reveal the conservation and variations in chloroplast genomes of these three species. The phylogenetic analysis was conducted by using the complete chloroplast genome sequences of various species to explore the phylogenetic position of these three species in Zygophyllaceae.

## Materials and methods

### Plant material, DNA extraction and sequencing

Fresh leaves of *T*. *mongolica*, *Z*. *xanthoxylon*, *and Z*. *fabago* were gathered from adult plants in Mengxi Town, Erdos City, Inner Mongolia Autonomous Region, in China. Total genomic DNA was extracted from the leaves utilizing the Plant Genomic DNA Kit (Tiangen Biotech Co., Beijing, China). The quality of DNA samples was assessed using a NanoDrop 2000 spectrophotometer (Nanodrop technologies, Wilmington, DE, USA) and agarose gel electrophoresis. Illumina paired-end DNA libraries with approximately 300 bp insert fragment were built using the NEBNext^®^ Ultra^™^ DNA Library Prep Kit and sequenced using an Illumina HiSeq2500.

### Chloroplast genome assembly and annotation

The raw data were processed by filtering adapter and low-quality reads using fastQC (http://www.bioinformatics.babraham.ac.uk/projects/fastqc/), then the clean data were used for genome assembly. GetOrganelle (https://github.com/Kinggerm/GetOrganelle) [21] and SPAdes (v. 3.9.0) [22] were used to assemble the clean data using the default parameter. The chloroplast genome assembly was then identified from the assembled sequences by align to *Tribulus terrestris* (NC_046758), *Arabidopsis* and tobacco chloroplast genomes [11, 23]. The online annotation tool DOGMA (http://dogma.ccbb.utexas.edu) [24] was utilized to annotate the protein-coding genes, tRNAscan-SE [25, 26] software was used to annotate the tRNA gene, and RNAmmer 1.2 server (http://www.cbs.dtu.dk/services/RNAmmer/) [27] was used for rRNA identification. The annotation results were edited using Sequin, and the resulting Sqn file was submitted to the GenBank database. The GenBank accession number of the chloroplast genomes of *T*. *mongolica*, *Z*. *xanthoxylon*, and *Z*. *fabago* were MK331720, MZ427318, and MK341052, respectively. The GenBank annotation files were submitted to Organellar Genome DRAW (OGDRAW) [28] to draw the visualized chloroplast genome map.

### Loss of *ndh* genes verification

To verify the loss of *ndh* genes in the chloroplast genomes of *T*. *mongolica*, *Z*. *xanthoxylon*, and *Z*. *fabago*, leaf DNA samples were extracted from tobacco and these three species and PCR experiments were performed on fragment *psaC*-*ndhE*-*ndhG*-*ndhI*-*ndhA*-*ndhH*-*rps15* and *rps7*-*ndhB*-*trnL-CAA* of the tobacco chloroplast genome and the fragment *psaC*-*rps15* and *rps7*-*trnL-CAA* of the chloroplast genomes in the three species. The PCR products were sequenced (BBI Life Sciences Co., Shanghai, China), and the sequencing results were spliced and compared with the references of the corresponding species. Details of gene fragments selected and primers in PCR were list in S1 Table.

### Genomic structure analysis

The Perl script MISA (https://webblast.ipk-gatersleben.de/misa/) [29] was used to detect microsatellites (mononucleotides, dinucleotides, trinucleotides, tetranucleotides, pentanucleotides, hexanucleotides) from three chloroplast genomes of Zygophyllaceae plants with the following thresholds: 10 repeat units of mononucleotide SSR, 6 repeat units of dinucleotide SSR, 5 repeat units of trinucleotide, tetranucleotide, pentanucleotide and hexanucleotide SSR. The online software REPuter (https://bibiserv.cebitec.uni-bielefeld.de/reputer) (University of Bielefeld, Bielefeld, Germany) [30] was utilized to predict the location and size of the repeat sequences, with the parameter set to spread the repeat copy at a percentage of at least 90% similarity, the minimum repeat size parameter was set as 30 bp.

### Identification of polymorphic loci

Multiple alignment was conducted among the chloroplast genomes of *T*. *mongolica*, *Z*. *xanthoxylon*, and *Z*. *fabago* after removing of IRA region utilizing MAFFT v7 [31]. The protein-coding regions and intergenic spacer regions were isolated from the alignment using Geneious R8.1 [32]. The nucleotide diversity values (Pi) and polymorphism of each sequence were calculated in DnaSP 6.12 [33] to investigate the polymorphic loci.

### Codon usage analysis

The distribution of codon usage was analyzed using the software CodonW (University of Texas, Houston, TX USA) [34] with the Relative synonymous codon usage (RSCU) value. RSCU value is an efficient index reflecting non-uniform usage of synonymous codons in a given coding sequence. In general, the RSCU value without any codon usage bias equals 1.00, and a RSCU below 1.00 indicates the relative probability of codon utilization is lower than expectation, just as the codon utilization frequency is higher than expectation while the RSCU may be above 1.00.

### Comparative genomics analysis

The comparison of gene order between chloroplast genomes of *T*. *mongolica* (MK331720), *Z*. *xanthoxylon* (MZ427318), *Z*. *fabago* (MK341052), *A*. *trichopoda* (NC_005086.1), *A*. *carambola* (NC_033350.1), *L*. *usitatissimum* (NC_036356.1), *E*. *novogranatense* (NC_030601.1), *G*. *maderense* (NC_029999.1), and *E*. *carvifolium* (NC_015083.1) was performed using MAUVE [35]. The online program mVISTA (http://genome.lbl.gov/vista/mvista/submit.shtml) [36] was utilized to find the divergence of chloroplast genomes of three species in Shuffle-LAGAN mode. The sequences were initially aligned according to MAFFT v7 [31] and manually adjusted based on BioEdit v7.0.9 [37].

### Phylogenetic analysis

Chloroplast genomes of 69 plant species were used to reconstruct the phylogenetic trees, and these species belong to Caryophyllales, Santalales, Vitales, Myrtales, Brassicales, Huerteales, Malvales, Sapindales, Oxalidales, Malpighiales, Celastrales, Rosales, Fagales, Cucurbitales, Zygophyllales, Fabales and Geraniales (S2 Table). The chloroplast genomes of 66 species were downloaded from the NCBI database to construct the phylogenetic tree using the Maximum Likelihood method. *A*. *trichopoda* were set as outgroup. The sequences of 50 shared protein-coding genes (*atpA*, *atpB*, *atpE*, *atpF*, *atpH*, *atpI*, *ccsA*, *cemA*, *matK*, *petA*, *petB*, *petD*, *petG*, *petL*, *petN*, *psaA*, *psaC*, *psaI*, *psaJ*, *psbA*, *psbB*, *psbC*, *psbD*, *psbE*, *psbF*, *psbH*, *psbI*, *psbJ*, *psbK*, *psbM*, *psbN*, *psbT*, *rbcL*, *rpl14*, *rpl16*, *rpl20*, *rpl22*, *rpl32*, *rpl36*, *rpoA*, *rpoB*, *rpoC1*, *rpoC2*, *rps3*, *rps4*, *rps8*, *rps14*, *rps18*, *ycf3*, *ycf4*) were extracted using TBtools V0.6669 [38] and aligned by MAFFT v7.427 [31]. After manual adjustment of the alignment, phylogenetic trees were rebuilt based on 50 common protein-coding gene sequences using MEGA X [39] software with 1000 bootstrap replicates.

## Results

### Genome content and organizations

**Fig 1:**
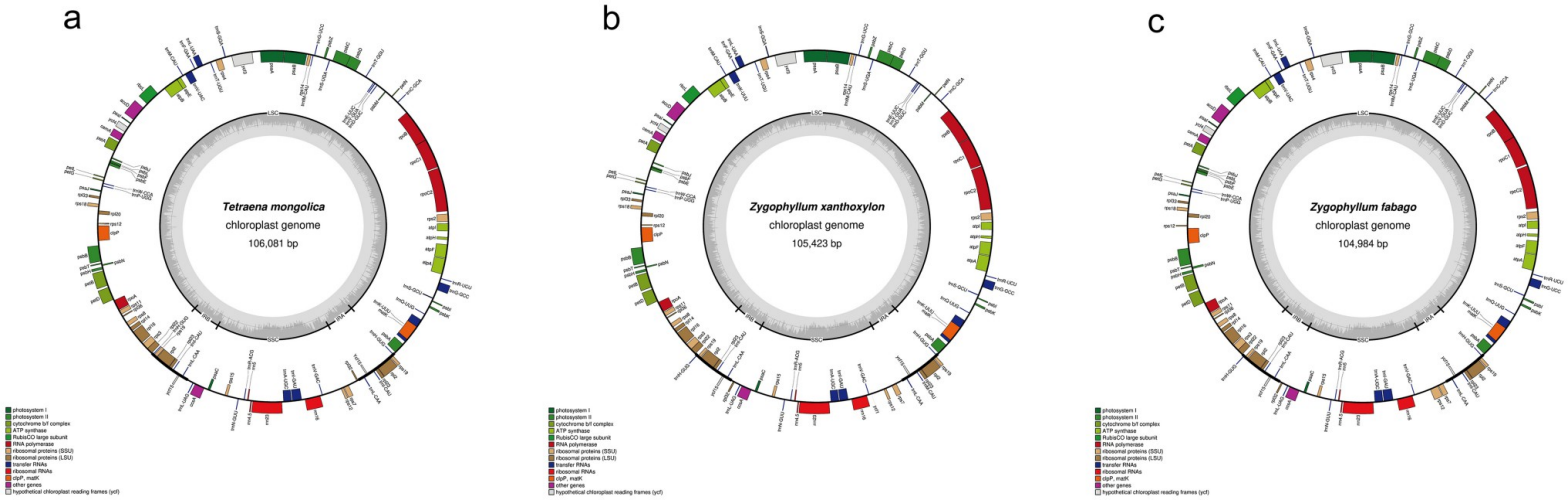
The complete chloroplast genome of *T*. *mongolica* (a), *Z*. *xanthoxylon* (b) and *Z*. *fabago* (c). The predicted genes are displayed and colors indicate functional classifications in the legend. The genes outside the circle are transcribed clockwise, whereas those inside the circle are transcribed counterclockwise. The inner gray circle describes the GC content. The large single copy (LSC), small single copy (SSC) and inverted repeat (IR) regions are marked in the inner circle.

Approximately 3 G, 3 G, and 7.1 G of 150 bp pair-end clean reads for *T*. *mongolica*, *Z*. *xanthoxylon*, and *Z*. *fabago*, respectively, were got from the Illumina sequencing, while the reads were assembled using GetOrganelle and SPAdes (Fig 1). The overall size of *T*. *mongolica*, *Z*. *xanthoxylon*, and *Z*. *fabago* chloroplast genomes are 106,081 bp, 105,423 bp, and 104,984 bp, respectively, which are significantly smaller than most of the plant chloroplast genomes. The chloroplast genomes of the three species show the typical quadripartite structure of angiosperm cpDNA, which consist of a large single copy (LSC) region of 80,291 bp in *T*. *mongolica*, 79,877 bp in *Z*. *xanthoxylon*, and 79,696 bp in *Z*. *fabago*, a small single copy (SSC) region of 17,162 bp in *T*. *mongolica*, 16,970 bp in *Z*. *xanthoxylon*, and 16,462 bp in *Z*. *fabago*, and a pair of inverted repeats (IRs) of 4,315 bp in *T*. *mongolica*, 4,288 bp in *Z*. *xanthoxylon*, and 4,413 bp in *Z*. *fabago*. The GC content of the chloroplast genomes are 33.7%, 34.06%, and 36.0%, respectively.

All the three chloroplast genomes encode 107 unique genes, including 70 protein-coding genes, 4 rRNA genes, and 33 tRNA genes (Tables 1 and 2). It is noteworthy that the rRNA genes located in IRs region in most higher plants present in the SSC region of the three Zygophyllaceae plants, and subsequently the copy number of rRNA genes change from 2 to 1. We compared the three Zygophyllaceae chloroplast genomes with that of *Amborella trichopoda*, which was thought to be the most primitive group of angiosperms, and the result showed that all the *ndh* genes encoding subunits of NADH oxidoreductase were lost in *T*. *mongolica*, *Z*. *xanthoxylon*, and *Z*. *fabago* which usually located in SSC and IRs. Moreover, *rps16*, *rpl12*, *ycf2* and *infA*, which were common in the chloroplast genomes of most angiosperms, lost in the chloroplast genomes of these three Zygophyllaceae plants.

**Table 1:** Genes identified from the chloroplast genomes of *T*. *mongolica*, *Z*. *xanthoxylon* and *Z*. *fabago*.

Category	Function	Gene names
Transcription and translation	Ribosomal proteins (LSU)	rpl2*, *rpl14*, *rpl16*, *rpl20*, *rpl22*, rpl23*, *rpl32*, *rpl33*, *rpl36*
	Ribosomal proteins (SSU)	*rps2*, *rps3*, *rps4*, *rps7*, *rps8*, *rps11*, *rps12*, *rps14*, *rps15*, *rps18*, rps19*
	RNA polymerase	*rpo*A, *rpo*B, *rpo*C1, *rpo*C2
	Ribosomal RNAs	*rrn4*.*5*, *rrn5*, *rrn16*, *rrn23*
	tRNA genes	*trnA-UGC*, *trnC-GCA*, *trnD-GUG*, *trnE-UUC*, *trnF-GAA*, *trnfM-CAU*, *trnG-GCC*, *trnG-UCC*, trnH-GUG*, trnI-CAU*, *trnI-GAU*, *trnK-UUU*, trnL-CAA*, *trnL-UAA*, *trnL-UAG*, trnM-CAU*, *trnN-GUU*, *trnP-UGG*, *trnQ-UUG*, *trnR-ACG*, *trnR-UCU*, *trnS-GCU*, *trnS-GGA*, *trnS-UGA*, *trnT-GGU*, *trnT-UGU*, *trnV-GAC*, *trnV-UAC*, *trnW-CCA*, *trnY-GUA*
Photosynthesis	ATP synthase	*atpA*, *atpB*, *atpE*, *atpF*, *atpH*, *atpI*
	Cytochrome b/f complex	*petA*, *petB*, *petD*, *petG*, *petL*, *petN*
	Photosystem I	*psaA*, *psaB*, *psaC*, *psaI*, *psaJ*
	Photosystem II	*psbA*, *psbB*, *psbC*, *psbD*, *psbE*, *psbF*, *psbH*, *psbI*, *psbJ*, *psbK*, *psbL*, *psbM*, *psbN*, *psbT*, *psbZ*
Other genes	Maturase	*matK*
	Envelop membrane protein	*cemA*
	Subunit Acetyl-CoA-Carboxylate	*accD*
	c-type cytochrome synthesis gene	*ccsA*
	ATP-dependent protease subunit gene	*clpP*
Unknown	Proteins of unknown function	*ycf1*, *ycf*3, *ycf*4, ycf15*

* Duplicate genes.

**Table 2:** Summary of major features of the three chloroplast genomes.

Item	*T*. *mongolica*	*Z*. *xanthoxylon*	*Z*. *fabago*
Total length (bp)	106,081	105,423	104,984
LSC length (bp)	80,291	79,877	79,696
SSC length (bp)	17,162	16,970	16,462
IR length (bp)	4,315	4,288	4,413
GC (%)	33.70	34.06	36.00
Total number of gene	107	107	107
No. of protein-coding genes	70	70	70
No. of rRNA	4	4	4
No. of tRNA	33	33	33

**Fig 2:**
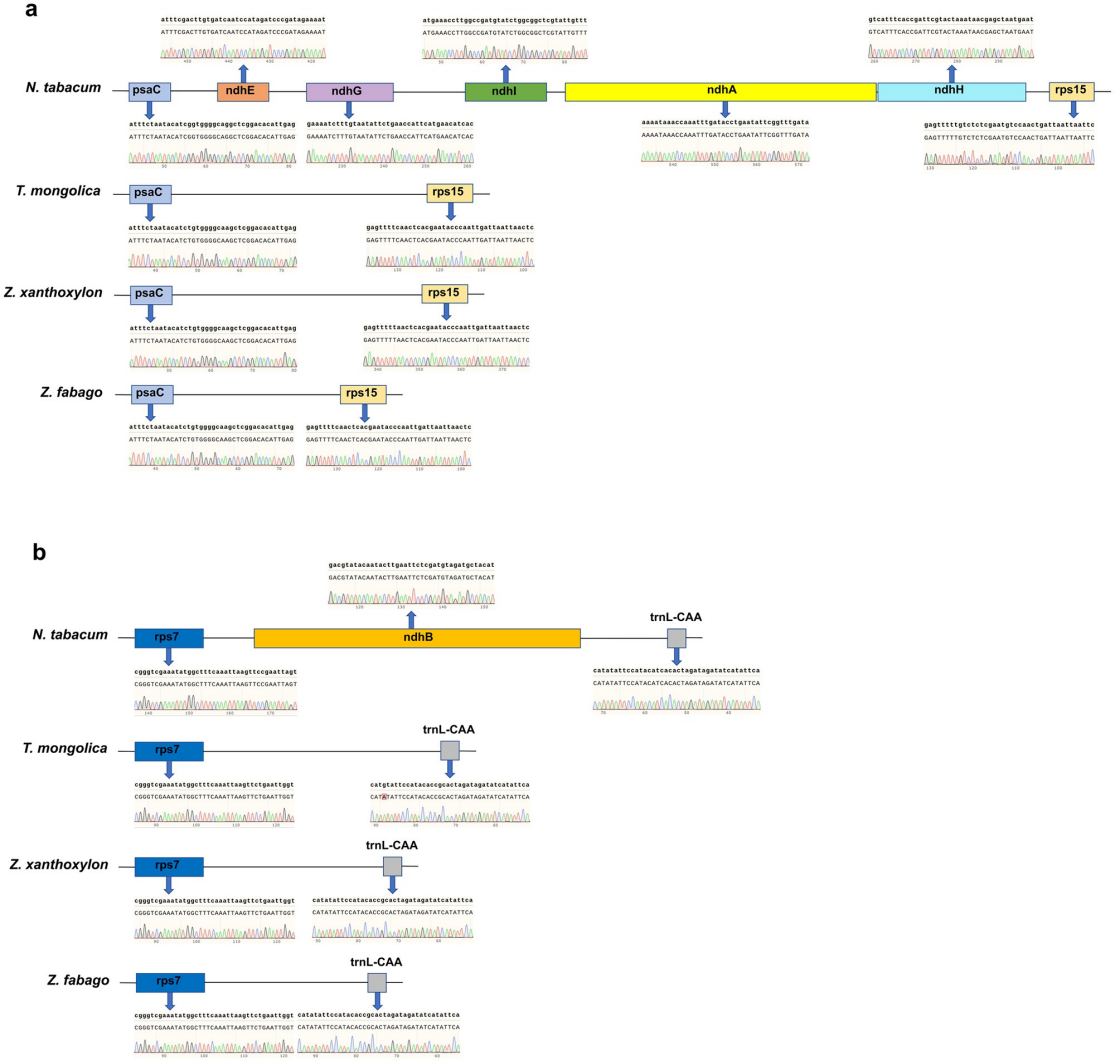
Comparison of sequencing results of PCR products among chloroplast genomes of tobacco, *T*. *mongolica*, *Z*. *xanthoxylon*, and *Z*. *fabago* to show the loss of *ndh* genes. Tobacco chloroplast genome as the reference. (a) Comparison of the fragment *psaC*-*ndhE*-*ndhG*-*ndhI*-*ndhA*-*ndhH*-*rps15* in tobacco and corresponding regions in the chloroplast genomes of *T*. *mongolica*, *Z*. *xanthoxylon*, and *Z*. *fabago*. (b) Comparison of the fragment *rps7*-*ndhB*-*trnL-CAA* in tobacco and corresponding regions in chloroplast genomes of *T*. *mongolica*, *Z*. *xanthoxylon*, and *Z*. *fabago*. The screenshots indicated by the blue arrows were excerpts from the PCR sequencing results of each gene.

To verify the loss of *ndh* genes in chloroplast genomes of these three species, utilizing tobacco as the reference, the gene fragment *psaC*-*ndhE*-*ndhG*-*ndhI*-*ndhA*-*ndhH*-*rps15* and *rps7*-*ndhB*-*trnL-CAA* located in SSC and IRA regions of tobacco chloroplast genome and the corresponding fragments in chloroplast genomes of *T*. *mongolica*, *Z*. *xanthoxylon*, and *Z*. *fabago* were selected for verification. The results showed that *ndhE*, *ndhG*, *ndhI*, *ndhA*, *ndhH* and *ndhB* genes were lost in the selected fragments of chloroplast genomes of *T*. *mongolica*, *Z*. *xanthoxylon*, and *Z*. *fabago* (Fig 2).

Introns play crucial roles in the regulation of gene expression [40–42]. In the chloroplast genomes of *T*. *mongolica*, *Z*. *xanthoxylon* and *Z*. *fabago*, 14 genes (*trnK-UUU*, *trnG-GCC*, *atpF*, *rpoC1*, *trnL-UAA*, *trnV-UAC*, *clpP*, *petB*, *petD*, *rpl16*, *rpl2*, *trnA-UGC*, *trnl-GAU* and *rpl2*) contain one intron, while one gene (*ycf3*) contain two introns (Table 3). The *trnK-UUU* gene, which contains the *matK* gene, has the largest intron with a length of 2544–2551 bp, while the length of other introns ranged from 455 bp to 943 bp.

**Table 3:** Intron-containing genes in chloroplast genomes of *T*. *mongolica*, *Z*. *xanthoxylon* and *Z*. *fabago*.

Gene	Location	Intron length in *T*. *mongolica* (bp)	Intron length in *Z*. *xanthoxylon* (bp)	Intron length in *Z*.*fabago* (bp)
*trnK-UUU*	SSC	2551	2544	2551
*trnG-GCC*	SSC	691	695	682
*atpF*	SSC	760	723	650
*rpoC1*	SSC	814	774	784
*ycf3* *	SSC	752, 757	736, 743	786, 747
*trnL-UAA*	SSC	460	455	465
*TrnV-UAC*	SSC	603	621	612
*clpP*	SSC	866	823	837
*petB*	SSC	749	793	791
*petD*	SSC	781	761	772
*rpl16*	SSC	943	855	940
*rpl2*	IRB	668	668	630
*trnA-UGC*	SSC	712	716	716
*trnl-GAU*	SSC	845	753	834
*rpl2*	IRB	668	671	666

* The gene has two introns.

### Repeat and SSRs analysis

Basic units made by 1–6 nucleotides repeated for several times form SSRs (Simple sequence repeats), which are widely utilized as molecular markers in molecular biology studies [43–45]. The types and distribution of SSRs in the chloroplast genomes of *T*. *mongolica*, *Z*. *xanthoxylon* and *Z*. *fabago* were predicted. The total number of SSRs detected in *T*. *mongolica*, *Z*. *xanthoxylon*, and *Z*. *fabago* were 76, 65, and 78. The most common SSRs were A or T mononucleotide repeats, accounting for 98.7%, 93.8%, and 97.4%, while no G or C repeats were predicted. In addition, mononucleotide and dinucleotides were identified in *T*. *mongolica*, *Z*. *xanthoxylon* and *Z*. *fabago*, respectively, and no trinucleotide, tetranucleotide, pentanucleotide, and hexanucleotide SSRs were predicted (Table 4). Most SSRs of *T*. *mongolica*, *Z*. *xanthoxylon* and *Z*. *fabago* were located in LSC regions (84.2%, 80.0%, and 84.6%, respectively), followed by SSC regions (13.2%, 10.8%, and 7.7%, respectively) (Table 5).

**Table 4:** Types and numbers of SSRs in chloroplast genomes of *T*. *mongolica*, *Z*. *xanthoxylon* and *Z*. *fabago*.

SSRs type	Repeat unit	Number in *T*. *mongolica* (bp)	Number in *Z*. *xanthoxylon* (bp)	Number in *Z*.*fabago* (bp)
Mono	A/T	75	61	76
Di	AT/TA	1	4	2
Tri	--	0	0	0
Tetra	--	0	0	0
penta	--	0	0	0
hexa	--	0	0	0
total	--	76	65	78

**Table 5:** The summary of SSRs distribution in different regions of three chloroplast genomes.

Species	LSC	IRA	SSC	IRB
*T*. *mongolica*	64	1	10	1
*Z*. *xanthoxylon*	52	3	7	3
*Z*. *fabago*	66	3	6	3

**Fig 3:**
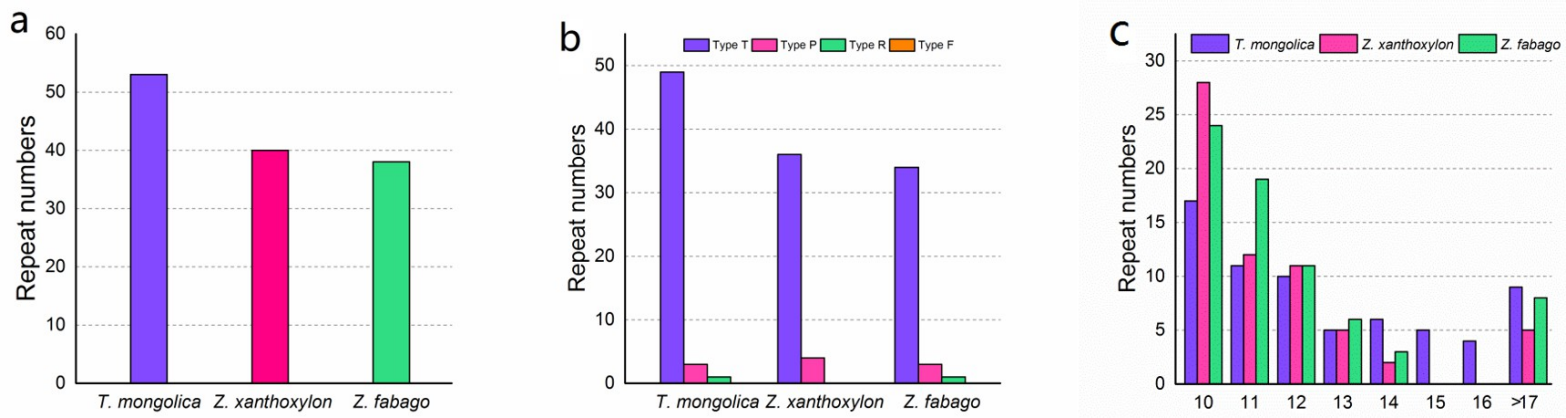
Long repeat sequences in chloroplast genomes of *T*. *mongolica*, *Z*. *xanthoxylon*, and *Z*. *fabago*. (a) Number of long repeats; (b) Number of different long repeats types; (c) Sequence length of long repeats.

We used REPuter [30] and Tandm Repeats Finder [46] to identify the palindrome repeats, forward repeats, reverse repeats, and tandem repeats of chloroplast genomes of *T*. *mongolica*, *Z*. *xanthoxylon*, and *Z*. *fabago* (Fig 3). A total of 53, 40, and 38 long repeats were detected in three chloroplast genomes (Fig 3a). The chloroplast genome of *T*. *mongolica* contained 49 tandem repeats, 3 palindrome repeats, and 1 reverse repeats. The chloroplast genome of *Z*. *xanthoxylon* contained 36 tandem repeats and 4 palindrome repeats, while the chloroplast genome of *Z*. *fabago* contained 34 tandem repeats, 3 palindrome repeats, and 1 reverse repeats (Fig 3b). In the three chloroplast genomes, long repeats with the length of 10 bp was the most common category, and then 11 bp and 12 bp categories (Fig 3c).

### Polymorphic loci analysis

**Fig 4:**
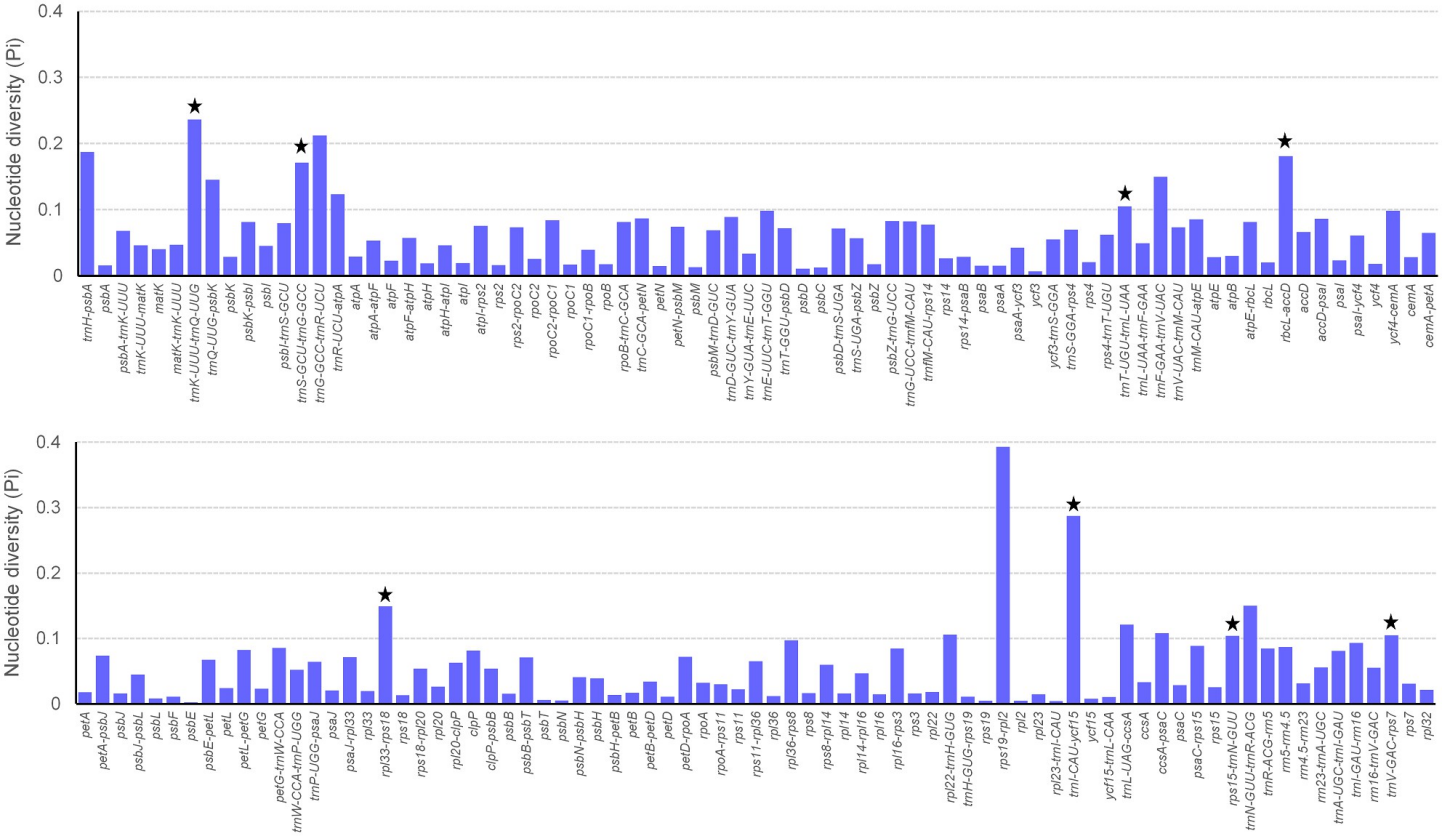
The nucleotide diversity values (Pi) of all regions. Regions with Pi = 0 are excluded and are not exhibited in the figure. The black starts show 8 polymorphic loci with the length>300 bp and Pi>0.1. The X-axis indicates chloroplast regions and the y-axis nucleotide diversity values (Pi).

The polymorphism of each region was exhibited (Fig 4). We selected 8 polymorphic regions with the length>300 bp and nucleotide diversity values (Pi)>0.1, *trnK-UUU-trnQ-UUG*, *trnS-GCU-trnG-GCC*, *trnT-UGU-trnL-UAA*, *rbcL-accD*, *rpl33-rps18*, *trnI-CAU-ycf15*, *rps15-trnN-GUU* and *trnV-GAC-rps7* (Table 6). All regions selected belonged to intergenic spacer regions, of which 5 presented in LSC region, 2 in SSC region and 1 in IR region.

**Table 6:** Polymorphic loci identified based on comparative analysis of chloroplast genomes of *T*. *mongolica*, *Z*. *xanthoxylon*, and *Z*. *fabago*.

Serial number	Region	Nucleotide diversity	Number of polymorphic sites	Alignment length	Conserved length
1	*trnI-CAU-ycf15*	0.28721	204	729	477
2	*trnK-UUU-trnQ-UUG*	0.23601	179	871	524
3	*rbcL-accD*	0.18088	105	777	387
4	*trnS-GCU-trnG-GCC*	0.17105	130	918	760
5	*rpl33-rps18*	0.14917	80	536	362
6	*trnT-UGU-trnL-UAA*	0.10503	103	1065	676
7	*trnV-GAC-rps7*	0.10490	180	2775	1716
8	*rps15-trnN-GUU*	0.10426	136	1114	908

### Codon usage

**Fig 5:**
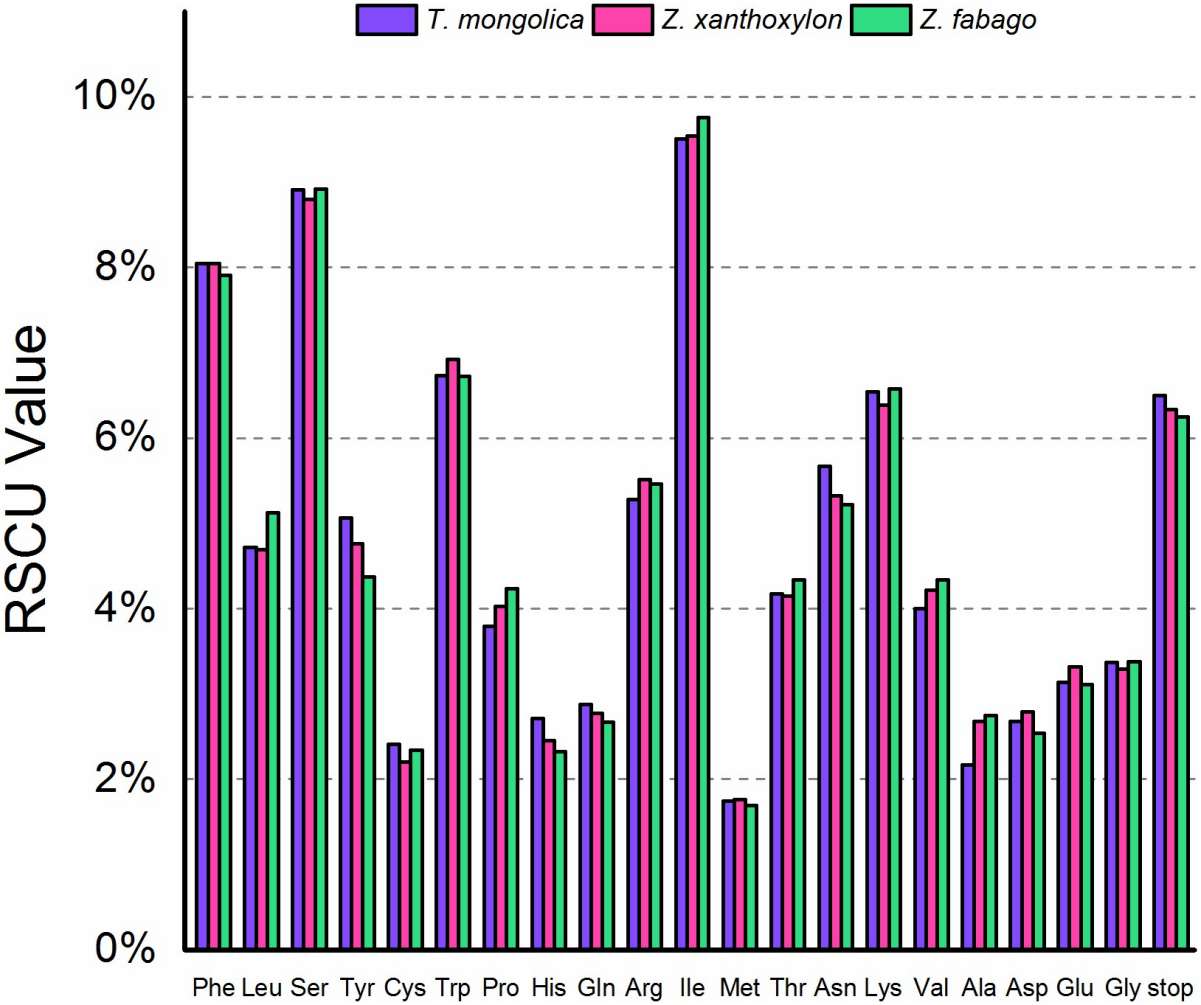
Proportion of codon preference in chloroplast genome of *T*. *mongolica*, *Z*. *xanthoxylon* and *Z*. *fabago*.

**Fig 6:**
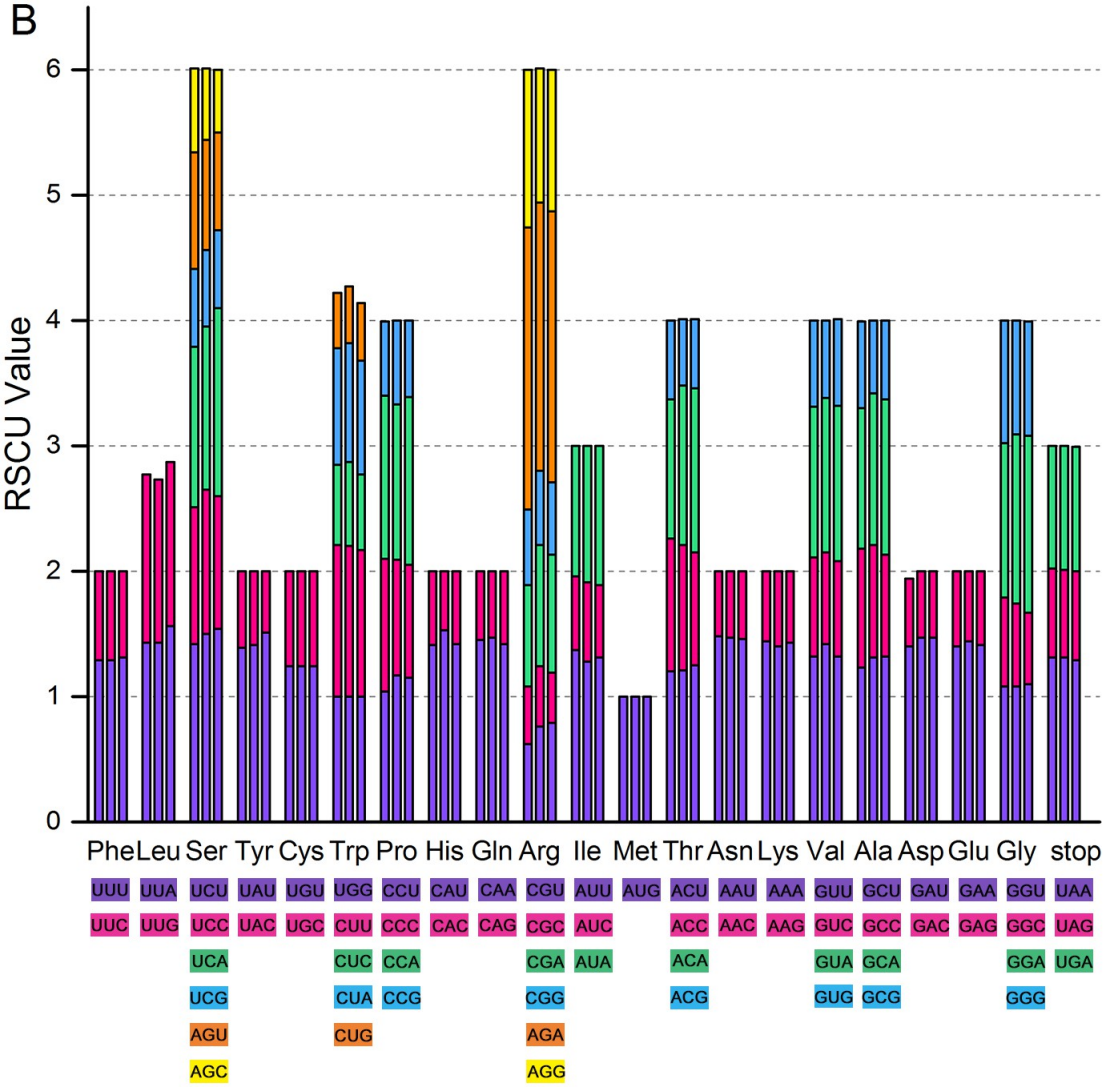
Codon content of 20 amino acids and stop codons in all protein-coding genes of chloroplast genomes of *T*. *mongolica*, *Z*. *xanthoxylon*, and *Z*. *fabago*.

Codon preference (codon usage bias) indicates the result of combined action of natural selection, species mutations, and genetic drift [47]. In the present study, according to the sequences of protein-coding genes, the frequency of codon usage of the chloroplast genomes of *T*. *mongolica*, *Z*. *xanthoxylon*, and *Z*. *fabago* was assessed (Figs 5 and 6). On the whole, the coding preferences of the three chloroplast genomes are very similar. All protein-coding genes of *T*. *mongolica*, *Z*. *xanthoxylon*, and *Z*. *fabago* consist of 35360, 35141, and 34994 codons, respectively. Among all these codons, isoleucine and methionine are the most frequently and the least frequently occurring amino acids in three chloroplast genomes. Specifically, there are up to 3362 (9.51%), 3362 (9.57%), and 3417(9.76%) isoleucine-encoding codons in chloroplast genomes of *T*. *mongolica*, *Z*. *xanthoxylon*, and *Z*. *fabago*, respectively; while there are 590 (1.67%), 619 (1.76%), and 615(1.76%) methionine-encoding codons in chloroplast genomes of *T*. *mongolica*, *Z*. *xanthoxylon*, and *Z*. *fabago*, respectively (Fig 5).

Relative synonymous codon usage analysis indicated that there was more than one synonym codon for almost all (except methionine) amino acids in the three chloroplast genomes, and the codons of UGG (tryptophan) and AUG (methionine) exhibited no usage bias (RSCU = 1) (Fig 6). About half of the codons have a RSCU value of >1.00 (30, 30, and 32 for the chloroplast genomes of *T*. *mongolica*, *Z*. *xanthoxylon*, and *Z*. *fabago*, respectively), and all codons with usage bias (RSCU>1) except CGU ended with A or U.

### Comparative genomics analysis

**Fig 7:**
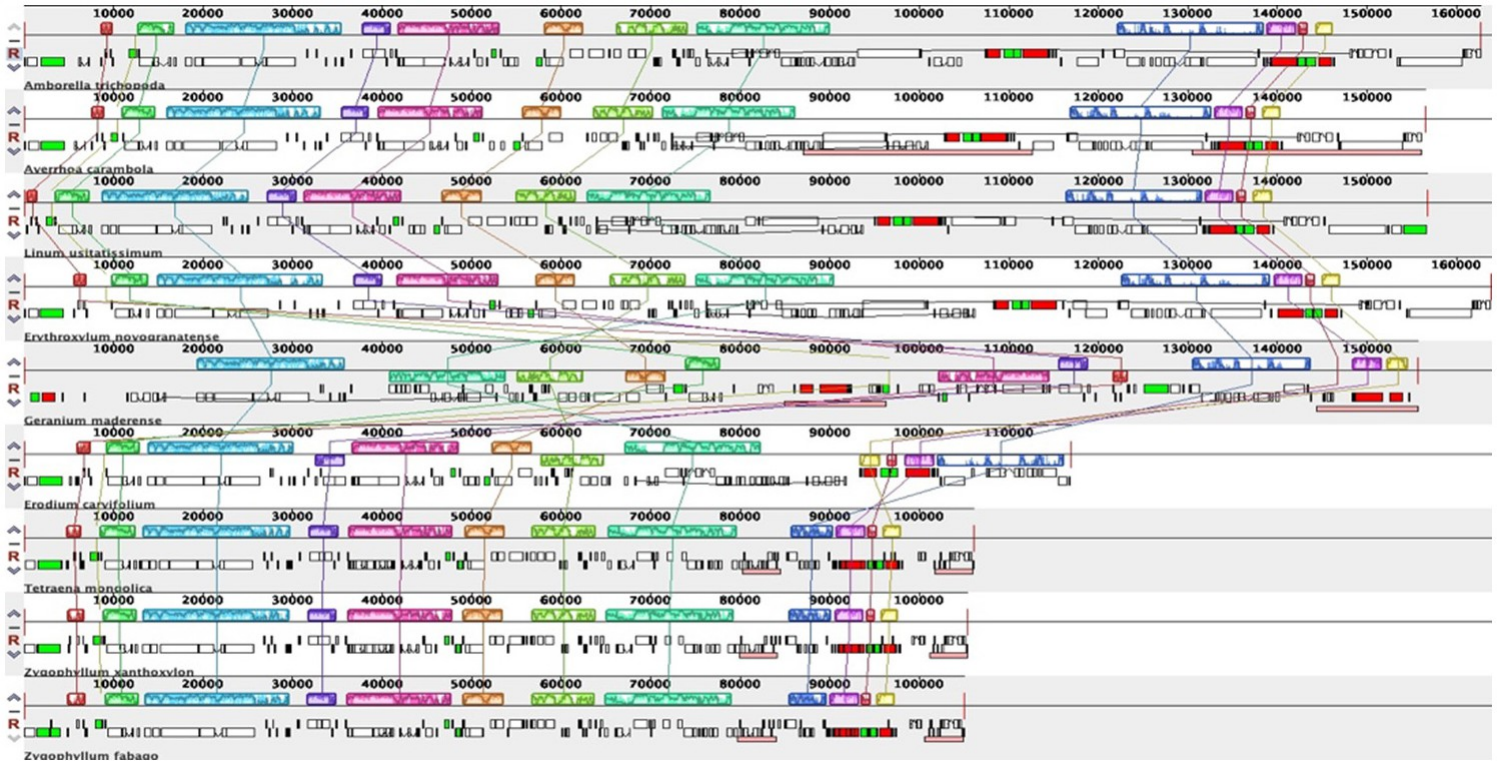
Gene order comparison of nine chloroplast genomes (*A*. *carambola*, *L*. *usitatissimum*, *E*. *novogranatense*, *G*. *maderense*, *E*. *carvifolium*, *T*. *mongolica*, *Z*. *xanthoxylon* and *Z*. *fabago*). *A*. *trichopoda* chloroplast genome as reference, utilizing MAUVE software. The boxes above the line indicate the gene sequences in clockwise direction and the boxes below the line indicate gene sequences in counterclockwise direction.

To detect gene loss and inversion, we compared the chloroplast genomes of the three Zygophyllaceae species with those of *Averrhoa carambola*, *Linum usitatissimum*, *Erythroxylum novogranatense*, *Geranium maderense*, and *Erodium carvifolium*, using MAUVE. The results pointed out that the size of the chloroplast genomes of the three Zygophyllaceae species were approximately (10–60) kb smaller than those of other species (Fig 7), and all 11 genes which encoded the subunits of NADH dehydrogenase (*ndh* gene) were lost from SSC and IRs. Moreover, the 4 rRNA that appeared in the IR region in most other plant were transferred to the SSC region in the three Zygophyllaceae species. In addition, compared with other species, there were no gene inversions in LSC region, SSC region, and IR region in the chloroplast genomes of the three Zygophyllaceae species.

**Fig 8:**
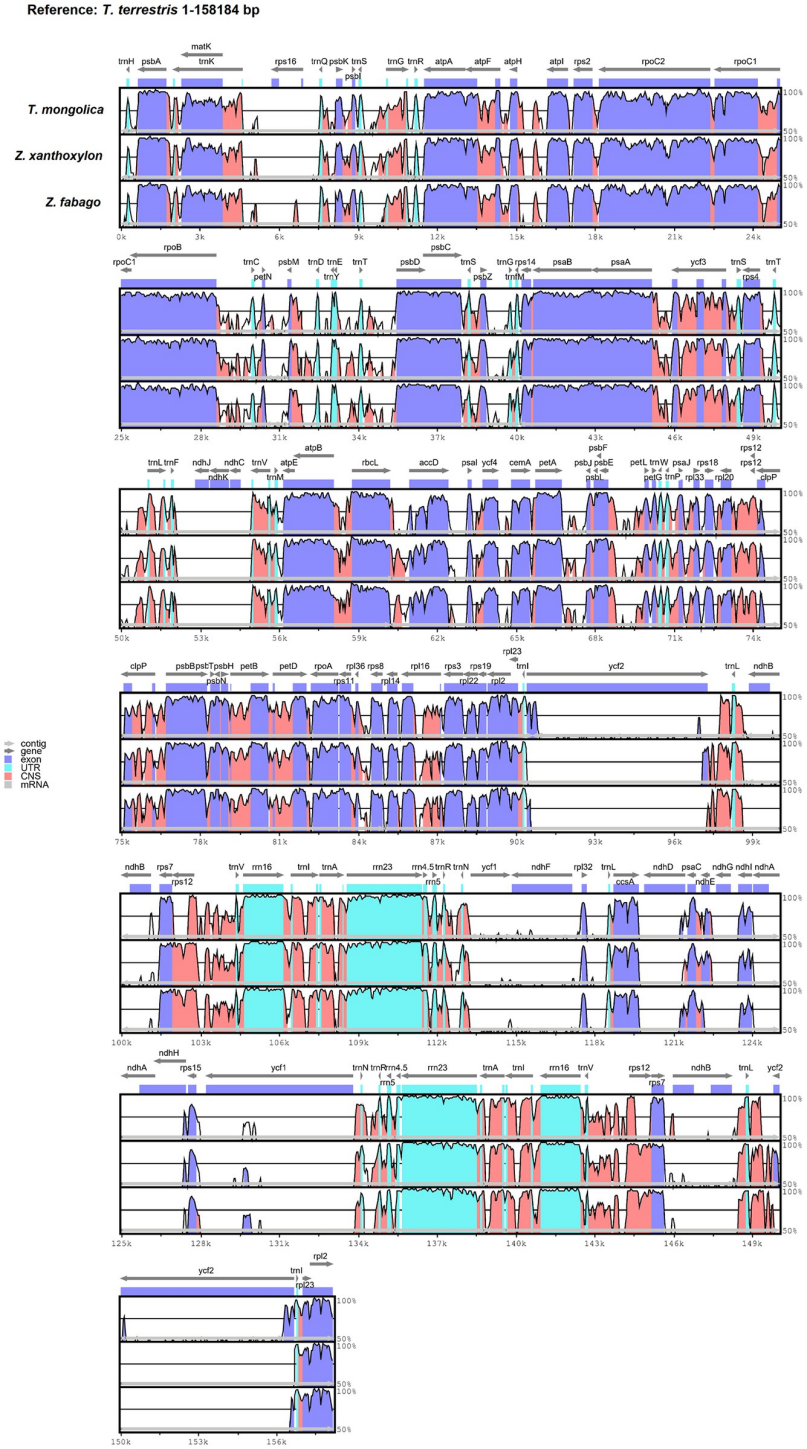
Comparison of the three chloroplast genomes utilizing *T*. *terrestris* as reference. Gray arrows and thick black lines above the alignment represent gene direction. Purple bars show exons, sky-blue bars show transfer RNA (tRNA) and ribosomal RNA (rRNA), red bars show non-coding sequences (CNS) and white peaks show the differences between chloroplast genomes. The y-axis indicates the identity percentage ranging from 50 to 100%.

In order to characterize genomic divergence between *T*. *mongolica*, *Z*. *xanthoxylon*, and *Z*. *fabago*, mVISTA software was employed to identify the divergent regions in the chloroplast genomes of the three Zygophyllaceae species, and *Tribulus terrestris* chloroplast genome was utilized as reference (Fig 8). The two IR regions were more conserved than LSC and SSC region, and the non-coding regions exhibited higher divergence than the coding regions. Moreover, the highest divergent regions in the three chloroplast genomes were detected in the intergenic spacer regions, including *trnK-trnQ*, *trnQ-psbK*, *trnS-trnG*, *trnG-trnR*, *trnR-atpA*, *atpF-atpH*, *trnR-atpA*, *rpoC1-rpoB*, *petN-psbM*, *trnE-trnT*, *trnG-UCC-trnfM-CAU*, *psbA-ycf3*, *trnT-trnL*, *trnF-trnV*, *atpB-rbcL*, *rbcL-accD*, *psbE-petL*, *rpl33-rps18*, *rps18-rpl20*, *rpl36-rps8*, *trnI-ycf15*, *psaC-rps15*, *rps15-trnN*, *trnN-trnR*, *trnV-rps12*, *rps7-rpl32*, and *rpl32-trnL*.

### Phylogenetic analysis

**Fig 9:**
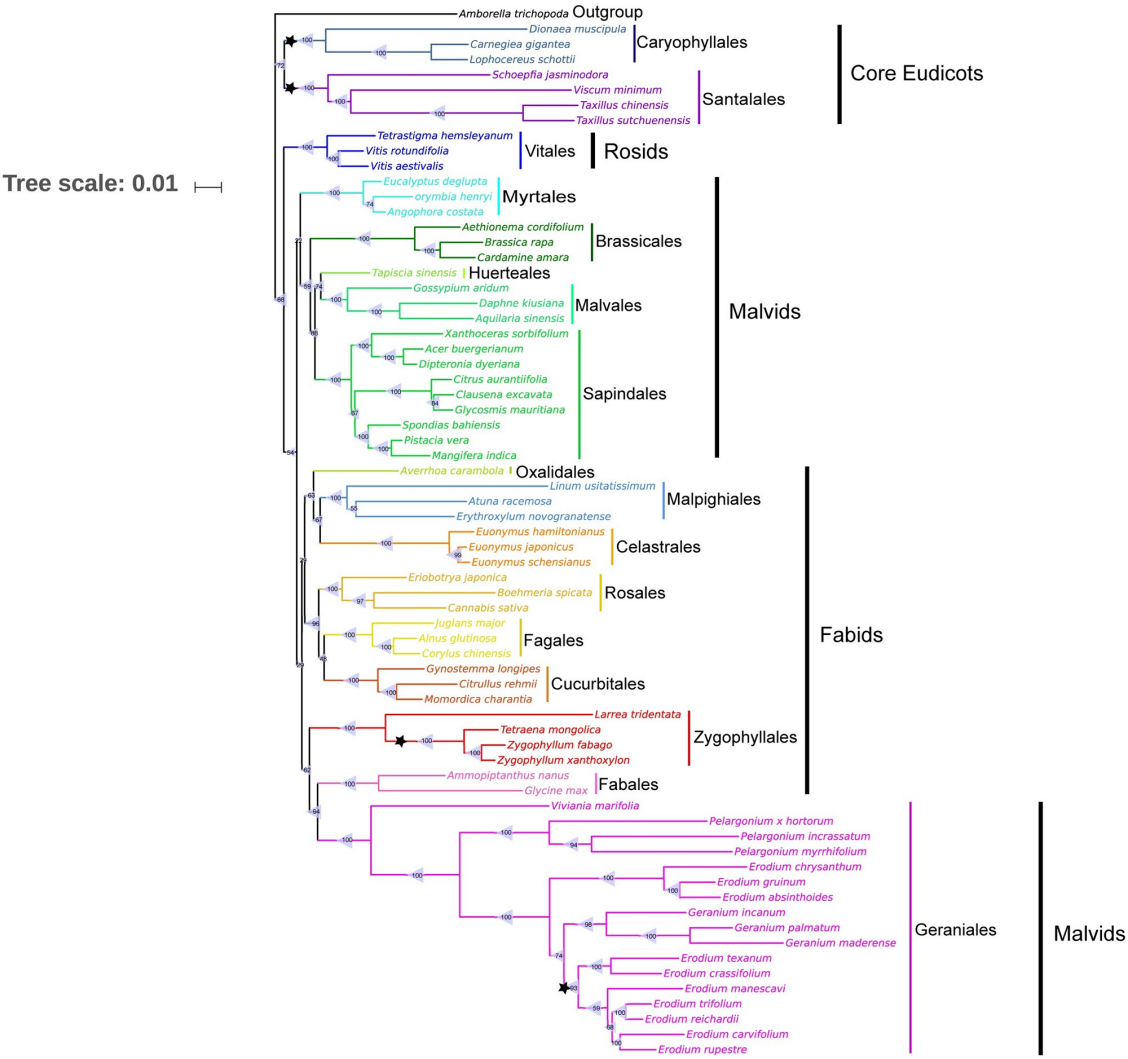
The phylogenetic tree of the sequences from 69 species, using Maximum Likelihood (ML) based on concatenated sequences of 50 genes implemented in MEGA X. *A*. *trichopoda* was set as the outgroup. Bootstrap supports were calculated from 1000 replicates. ▲ Represent the bootstrap value. ★ Represent the missing evolutionary branch of the 11 gene.

To investigate the phylogenetic status of the three Zygophyllaceae species in angiosperms and their interspecific relationships, 50 protein-coding genes from 69 plant species were phylogenetically analyzed using MEGA X software (Fig 9). All the plants chosen belong to the Core Eudicots branch according to the APG classification [6, 7, 48, 49]. The results indicated that Caryophyllales and Santalales were early-divergent angiosperms, and order Vitales was the earliest divergent clade of Rosids. Of Malvids and Fabids clades, Myrtales, Geraniales, and Zygophyllales were early evolutionary groups. As expected, the three Zygophyllaceae species were clustered in the Fabids clade together with Oxalidales, Malpighiales, Celastrales, Rosales, Fagales, Cucurbitales, and Fabales. But unexpected, the four Zygophyllales plants were clustered in one branch with Geraniales and Fabales, considered that Geraniales was classified in Malvids according to the latest APG classification. In Zygophyllaceae, *Z*. *xanthoxylon* and *Z*. *fabago* formed a monophyletic branch with 100% bootstrap value, and the branch was sister clade to the genus *Tetraena*.

### IR expansion and contraction

**Fig 10:**
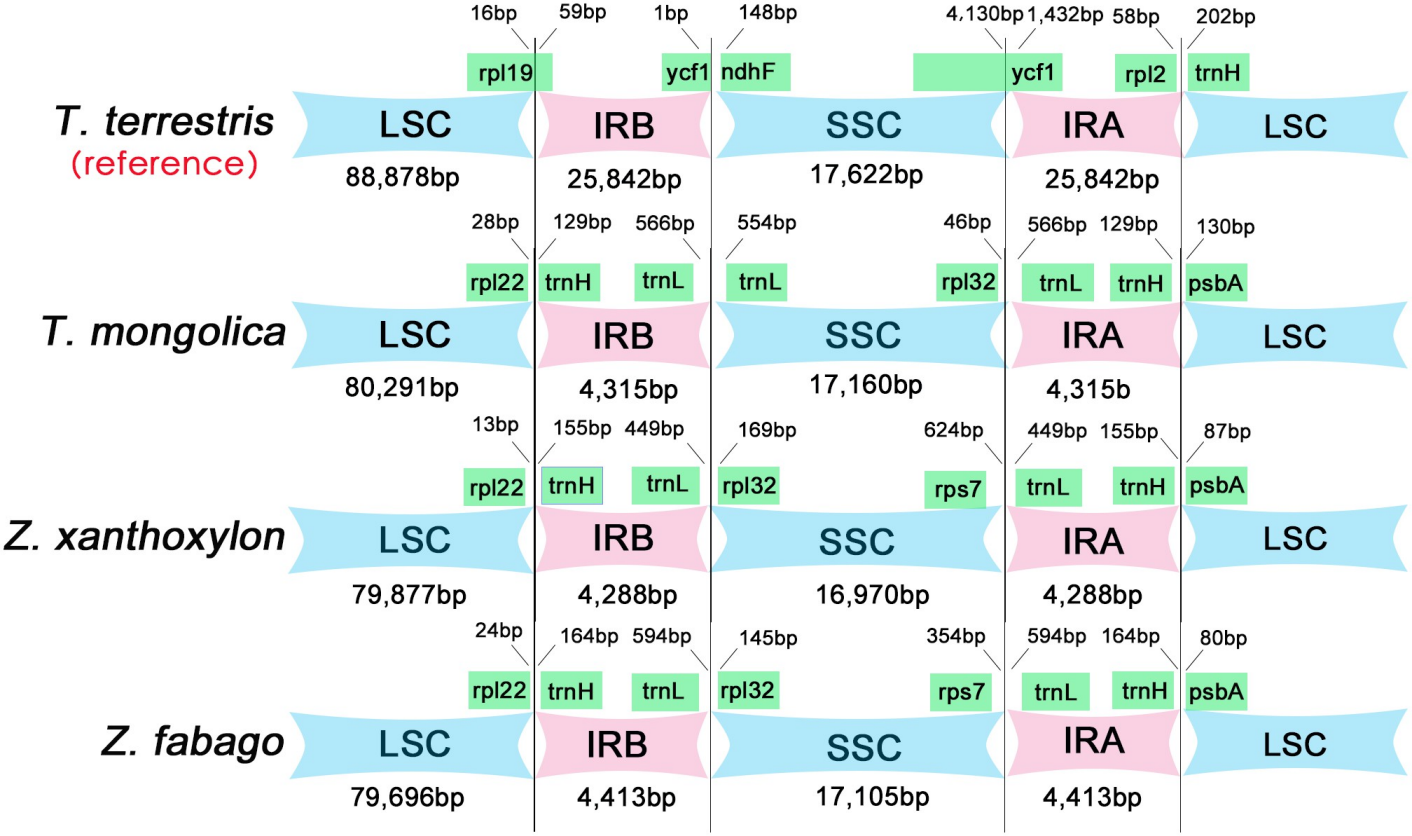
IR expansion and contraction in three chloroplast genomes. Gene names are shown in boxes, and genes lengths in the corresponding regions are marked above the boxes. These features are not to scale.

Although the IR region is thought to be the most conserved region in chloroplast genome, the contraction and expansion of the IR region boundary is a common phenomenon in the evolution of the chloroplast genome and the main cause of the chloroplast genome size alteration [50–52]. Here, we conducted a comparative analysis of the IR/LSC and IR/SSC boundary regions of *T*. *terrestris*, *T*. *mongolica*, *Z*. *xanthoxylon*, and *Z*. *fabago* (Fig 10). In these three chloroplast genomes of *T*. *mongolica*, *Z*. *xanthoxylon*, and *Z*. *fabago*, no pseudogenes and genes crossing the border were found. The boundary was between *rpl22* and *trnH-GUG* on the IRB/LSC side, and between *trnH-GUG* and *psbA* on the IRA/LSC side. In *T*. *mongolica*, the boundary of IRB/SSC was located between *trnL-CAA* and *trnL-UAG*, and the boundary of IRA/SSC was between *rpl32* and *trnL-CAA*. In *Z*. *xanthoxylon* and *Z*. *fabago*, the boundary of IRB/SSC was located between *rpl32* and *trnL-CAA*, and the boundary of IRA/SSC was between *rps7* and *trnL-CAA*.

Specifically, in the IR region of *T*. *mongolica*, *Z*. *xanthoxylon* and *Z*. *fabago*, *trnH-GUG* deviates from the IR/LSC boundary by 129 bp, 155 bp, and 164 bp, respectively. *trnL-CAA* is 566 bp, 449 bp, 594 bp, respectively, from the IR/SSC boundary. The gene *rpl22* located in LSC, which was 13–28 bp from the IRB/LSC border, similarly, the gene *psbA* deviated from the IRA/LSC by 80–130 bp. Among the three species, the genes close to the IR/SSC border in SSC were different. In *T*. *mongolica*, *trnL-UAG* was 554bp from IRB/SSC boundary, and *rpl32* was 46bp from IRA/SSC boundary. In both *Z*. *xanthoxylon* and *Z*. *fabago*, *rpl32* and *rps7* located close to the border of IRB/SSC and IRA/SSC.

## Discussion

The sizes of the three Zygophyllaceae chloroplast genomes are significantly shorter than those of most angiosperms. In majority of angiosperms, the chloroplast genomes are 120–160 kb in length, while the sizes of the chloroplast genomes of *T*. *mongolica*, *Z*. *xanthoxylon*, and *Z*. *fabago* range from 104 to 106 kb. The LSC regions of most angiosperms are generally about 80–90 kb in length, while the SSC regions are about 16–27 kb in length, and the size of two IRs are approximately 20–28 kb. Compared with most angiosperms, the sizes of LSC and SSC of *T*. *mongolica*, *Z*. *xanthoxylon*, and *Z*. *fabago* don’t change significantly, and the most conspicuous change is occurred in two IRs reduced by about 16–24 kb in size. Thus, the reduced sizes of chloroplast genomes of these three Zygophyllaceae species are mainly associated with the shrinkage of IRs.

Although the chloroplast genome is highly conservative, several chloroplast genomes are significantly smaller than that of most other plants, and some of them are listed in S3 Table. The most common reports of small chloroplast genomes came from studies of chloroplast genomes in parasitic plants, including *Taxillus chinensis* and *T*. *sutchuenensis* in Loranthaceae of Santalales [53], *Epifagus virginiana* in Orobanchaceae of Lamiales [54], *Cuscuta chinensis* and *C*. *japonica* in Convolvulaceae of Solanales [55]. Smaller chloroplast genomes were also found in some gymnosperms such as *Welwitschia mirabilis* in Welwitschiaceae of Welwitschiales [56], and *Gnetum ula* in Gnetaceae of Gnetales [57]. In non-parasitic angiosperms, the chloroplast genome with the size smaller than 130 kb was rarely reported except *Astragalus membranaceus*, whose chloroplast genome was approximately 124 kb, partly due to the loss of an IR [58]. The shrinkage of chloroplast genomes of the other plant species were associated with significant reduction in size of SSCs, for example, the SSCs of chloroplast genomes of the parasitic plants in S3 Table were less than half of that in tobacco [59], a classical angiosperm chloroplast genome. In the three Zygophyllaceae species, the sizes of LSC and SSC decrease slightly, but the lengths of IRs decrease dramatically. Thus, the three Zygophyllaceae species could be utilized as novel models to investigate the evolution of chloroplast genome structure and size.

Comparison of three Zygophyllaceae chloroplast genomes with those of other plant species reveal that, 4 rRNA genes usually presented in IRs are located in SSC region in these three chloroplast genomes, and thus leading to the reduction of the copy number of rRNA genes. In addition, it had been reported that due to the contraction and expansion of IR regions in the chloroplast genome of *Pothos scandens* in Araceae, some genes which existed in IR regions transferred to the LSC region becoming single copy and most of genes which appeared in SSC region transferred to the IR regions turning into duplicated, resulting in the change of gene numbers and the increased size of LSC region and the decreased size of SSC region [60]. Different from our study, although the IR regions had contracted and expanded, there was no loss of genes and no significant change in the size of the chloroplast genome in *Pothos scandens*. Similar to our observation, previous studies had reported rRNA gene displacement in *Erodium* species [61]. And all *ndh* genes usually located in SSC and IRs region encoding subunits of NADH oxidoreductase are lost. Moreover, *rps16*, *rpl12*, *ycf2* and *infA*, which are common in most angiosperm chloroplast genomes, are lost in chloroplast genomes of *T*. *mongolica*, *Z*. *xanthoxylon*, and *Z*. *fabago*. All above may be the possible reasons for the size reduction of IRs region. In addition, the NADH dehydrogenase complex in plant plastids are involved in photosynthesis in response to environmental stress. Although very uncommon, the *ndh* gene losses or pseudogenization are widespread phenomena in chloroplasts of different lineages of seed plants which are photoautotrophic [62]. The phenomenon had been reported that the *ndh* genes of plant plastid were specifically lost and NDH subunits which were nuclear-encoded were expression in Pinaceae [57], Orchidaceae [63], gnetophytes [64] and Geraniales [61]. Adaptation to the environment is especially critical for plants grow in barren soil in the arid and semi-arid regions. The current result reveals the loss of 11 *ndh* genes in these chloroplast genomes of *T*. *mongolica*, *Z*. *xanthoxylon*, and *Z*. *fabago*, and it is not certain whether *ndh* genes encoded by plastid have been lost completely or moved to cell nucleus functionally for the three Zygophylloideae species, which deserves to be discussed.

Previously reports had shown that losses of plastid-encoded *ndh* genes in Pinaceae possibly occurred before the divergence of this lineage (140 MYA) [57, 65]. The most recent losses of plastid-encoded *ndh* genes were found in a long divergent branch with 13 species in *Erodium* which had been supposed to predate the divergence of this branch (3 MYA) [61, 62]. A more recent phenomenon of pseudogenization of 4 *ndh* genes in genus *Melianthus* of Geraniales [66]. This branch was found to have diversified about 2 MYA and preserved some translatable sequences in the plastome [62]. In our study, *T*. *mongolica* was from the genus *Tetraena* of Zygophylloideae, and *Z*. *xanthoxylon* and *Z*. *fabago* belonged to the genus *Zygophyllum* of Zygophylloideae. All 11 plastid-encoded *ndh* genes were loss in the three species. However, the *ndh* genes were intact in chloroplasts of *Larrea tridentata* of Larreoideae and *Tribulus terrestris* of Tribuloideae. Subfamily Larreoideae and Tribuloideae were classified into Zygophyllaceae. It might suggest that the loss of plastid-encoded *ndh* genes in the three species involved in our study had possibly occurred ahead of the divergence of subfamily Zygophylloideae (38 MYA) [67]. However, due to the limited number of species chosen in our study, more species from Zygophyllaceae and Zygophylloideae could be added in subsequent studies which is helpful to further explore the loss of *ndh* genes and the function of NADH complex in Zygophyllaceae.

In prior studies, the correlations of repeats, SNPs and InDels were analyzed in chloroplast genomes of Malvaceae [68]. It was shown fluctuations in correlations at the family level, the subfamily level and the genus level in quantitative researches. While up to 90% of repeats and SNPs were simultaneous, and 52%-72% of repeats contained InDels at the family and subfamily level in qualitative studies. And it was hypothesized that the correlations among mutation events might be a usual feature in plant chloroplast genomes. This showed the important role of repeats in the generation of SNPs and InDels. 10 polymorphic loci were identified in chloroplast genomes of *Blumea* species, among which 5 regions were concurrent with repeats [69]. In our current study, we identified 8 polymorphic loci, and 7 were existed in the regions where repeats emerged except *rps15-trnN-GUU*. The co-occurrence proportion of repeats and polymorphic loci was as high as 87.5%. This result also supported the view that repeats could be utilized to identify the polymorphic loci for future researches on phylogeny and taxonomic status of plant.

Phylogenetic trees based on 50 common protein-coding genes in the chloroplast genomes of 69 plant species provide crucial molecular evidence for exploring the phylogenetic status of the three Zygophyllaceae species. Considered that Zygophyllaceae had been classified in the order of Geraniales in *Flora Reipublicae Popularis Sinicae* [4] and *Flora of China* [5], our results support the latest taxonomic classification of Zygophyllaceae described in APG IV in which Zygophyllaceae belongs to Fabids rather than Malvids. *T*. *mongolica*, *Z*. *xanthoxylon* and *Z*. *fabago* are clustered into a single branch with *Larrea tridentata*, which is another species in Zygophyllaceae, and the four Zygophyllaceae species are clustered in the Fabids clade together with Oxalidales, Malpighiales, Celastrales, Rosales, Fagales, Cucurbitales, and Fabales. Our phylogenetic analyses also reveal the close relationship between *Z*. *fabago* and *Z*. *xanthoxylon*, and support the incorporation of the *Z*. *xanthoxylon* into the genus *Zygophyllum*.

At the same time, our phylogenetic analysis also raises some new speculations on the evolutionary status of Zygophyllaceae and other related taxonomic branch, which need to be investigated further. First, our results show that Zygophyllales is clustered in a small branch with Fabales, but not with other orders in Fabids like Oxalidales, Malpighiales, and Rosales, indicating a closer relationship between Zygophyllales and Fabales which is not reported in previous reports. Second, it is surprisingly to find Zygophyllales of Fabids are clustered in a single clade with many plant species in Geraniales, which are classified into Malvids according to APG IV [7]. Our results raise a possibility that at least part of species in Geraniales belong to Fabids instead of Malvids, just as Zygophyllales was once classified in Malvids and is now classified in Fabids.

It should be noted that in our study the phylogenetic tree was constructed based on 69 species belonging to 51 genera and 30 families, including plants from Oxalidales, Malpighiales, Celastrales, Rosales, Fagales, Cucurbitales and Fabales which were also classified into Fabids like Zygophyllaceae, and species from Malvids with disputed classification. Four species from two subfamilies (six in total) of Zygophyllaceae, among which three species from two genera (six in total) of Zygophylloideae, were chosen in this study. The three species were *T*. *mongolica*, *Z*. *xanthoxylon*, and *Z*. *fabago* with significant shortage in size of the chloroplast genomes which were concerned in our study. Based on the limited number of species selected, future research could consider more species of Zygophyllaceae to conduct more detailed phylogeny analysis. It will be helpful to explore the phylogenetic status and evolution of Zygophyllaceae.

In brief, we assemble the whole chloroplast genomes of *T*. *mongolica*, *Z*. *xanthoxylon*, and *Z*. *fabago*. Our study reveals the unusual reduction of the three chloroplast genomes, especially IR regions, and the loss of 11 genes cording subunits of NADH dehydrogenase in SSC and IRs region. Comparative genomics identify the genetic variation between the chloroplast genomes of the three Zygophyllaceae species and other plant species. Phylogenetic analysis according to 50 common protein-coding genes of 69 plant chloroplast genomes support current understanding of the phylogenetic status of Zygophyllaceae.

## Supporting information

S1 Table Details of gene fragments selected and corresponding primers in PCR to verify the loss of *ndh* genes. (DOCX)

S2 Table Chloroplast genomes of the sixty-nine plant species used for phylogenetic analysis. (DOCX)

S3 Table Reported chloroplast genomes with size smaller than 130 kb and tobacco chloroplast genome. (DOCX)
