# Supplementary material for: The complete chloroplast genome sequencing analysis revealed an unusual IRs reduction in three species of subfamily Zygophylloideae
Source: PLoS One. 2022 Feb 2;17(2):e0263253. doi: 10.1371/journal.pone.0263253 (PMC8809528; doi:10.1371/journal.pone.0263253)
Supplement: S1 Table — (DOCX) [file pone.0263253.s001.docx]

**S1 Table.** **Details of gene fragments selected and corresponding primers in PCR to verify the loss of *ndh* genes.**

|  | *Nicotiana tabacum* | | | | *Tetraena mongolica* | | | | *Zygophyllum xanthoxylon* | | | | *Zygophyllum fabago* | | | |
| --- | --- | --- | --- | --- | --- | --- | --- | --- | --- | --- | --- | --- | --- | --- | --- | --- |
| **Genes** | **Start** | **End** | **Length** | **Location** | **Start** | **End** | **Length** | **Location** | **Start** | **End** | **Length** | **Location** | **Start** | **End** | **Length** | **Location** |
| *psaC* | 119146 | 119391 | 246 | SSC | 86719 | 86964 | 246 | SSC | 86587 | 86832 | 246 | SSC | 86562 | 86807 | 246 | SSC |
| *ndhE* | 119652 | 119957 | 306 | SSC | － | － | － | － | － | － | － | － | － | － | － | － |
| *ndhG* | 120181 | 120711 | 531 | SSC | － | － | － | － | － | － | － | － | － | － | － | － |
| *ndhI* | 121108 | 121611 | 504 | SSC | － | － | － | － | － | － | － | － | － | － | － | － |
| *ndhA* | 121696 | 123935 | 2240 | SSC | － | － | － | － | － | － | － | － | － | － | － | － |
| *ndhH* | 123937 | 125118 | 1182 | SSC | － | － | － | － | － | － | － | － | － | － | － | － |
| *rps15* | 125230 | 125493 | 264 | SSC | 88465 | 88740 | 276 | SSC | 88298 | 88573 | 276 | SSC | 87904 | 88179 | 276 | SSC |
| *rps7* | 142622 | 143089 | 468 | IRA | 100203 | 100670 | 468 | SSC | 100045 | 100512 | 468 | SSC | 99751 | 100218 | 468 | SSC |
| *ndhB* | 143369 | 145580 | 2212 | IRA | － | － | － | － | － | － | － | － | － | － | － | － |
| *trnL-CAA* | 146120 | 146200 | 81 | IRA | 102332 | 102412 | 81 | IRA | 101584 | 101666 | 83 | IRA | 101165 | 101245 | 81 | IRA |
| **Primer names** | | | | | **Sequences (5’ to 3’)** | | | | | | | | | | | |
| *rps7*-*trnL-CAA*-1F | | | | | ccgtcaaagcaagacgtgtagg | | | | | | | | | | | |
| *rps7*-*trnL-CAA*-1R | | | | | cctccacgctatttagcacgaga | | | | | | | | | | | |
| *rps7*-*trnL-CAA*-2F | | | | | ccccactcggaccaagacagaa | | | | | | | | | | | |
| *rps7*-*trnL-CAA*-2R | | | | | tgcctgccatccacaccagaa | | | | | | | | | | | |
| *rps7*-*trnL-CAA*-3F | | | | | ggctctctctttagccctatgtctc | | | | | | | | | | | |
| *rps7*-*trnL-CAA*-3R | | | | | acgagattttgagtctcgcgtg | | | | | | | | | | | |
| *psaC*-*rps15*-1F | | | | | cttacaaccaacacagtcctctgt | | | | | | | | | | | |
| *psaC*-*rps15*-1R | | | | | cacgcaaatcgtttacctgtaac | | | | | | | | | | | |
| *psaC*-*rps15*-2F | | | | | aacgttccgatgtgatcaatttttc | | | | | | | | | | | |
| *psaC*-*rps15*-2R | | | | | acggagacttacttcacatttagaa | | | | | | | | | | | |
| *psaC*-*rps15*-3F | | | | | gcaaattgataaaacctggtgggc | | | | | | | | | | | |
| *psaC*-*rps15*-3R | | | | | ttcagatacgtcatggtacggg | | | | | | | | | | | |
| *psaC*-*rps15*-4F | | | | | acgagctacagcaattgcacc | | | | | | | | | | | |
| *psaC*-*rps15*-4R | | | | | ggccctttttctgagaaaggagaa | | | | | | | | | | | |
| *psaC*-*rps15*-5F | | | | | cccctttttgcttttgcaattagat | | | | | | | | | | | |
| *psaC*-*rps15*-5R | | | | | tctcataggagatcagagcgt | | | | | | | | | | | |
