# Supplementary material for: The complete chloroplast genome sequencing analysis revealed an unusual IRs reduction in three species of subfamily Zygophylloideae
Source: PLoS One. 2022 Feb 2;17(2):e0263253. doi: 10.1371/journal.pone.0263253 (PMC8809528; doi:10.1371/journal.pone.0263253)
Supplement: S2 Table — (DOCX) [file pone.0263253.s002.docx]

**S2 Table. Chloroplast genomes of the sixty-nine plant species used for phylogenetic analysis.**

| **Order** | **In/Out group** | **Category** | **Order** | **Species** | **Accession** |
| --- | --- | --- | --- | --- | --- |
| 1 | Ingroup | Superasterids | Caryophyllales | *Dionaea muscipula* | NC_035417.1 |
| 2 | Ingroup | Superasterids | Caryophyllales | *Carnegiea gigantea* | NC_027618.1 |
| 3 | Ingroup | Superasterids | Caryophyllales | *Lophocereus schottii* | NC_041727.1 |
| 4 | Ingroup | Superasterids | Santalales | *Schoepfia jasminodora* | NC_034228.1 |
| 5 | Ingroup | Superasterids | Santalales | *Viscum minimum* | NC_027829.1 |
| 6 | Ingroup | Superasterids | Santalales | *Taxillus chinensis* | NC_036306.1 |
| 7 | Ingroup | Superasterids | Santalales | *Taxillus sutchuenensis* | NC_036307.1 |
| 8 | Ingroup | Rosids | Vitales | *Tetrastigma hemsleyanum* | NC_029339.1 |
| 9 | Ingroup | Rosids | Vitales | *Vitis rotundifolia* | NC_023790.1 |
| 10 | Ingroup | Rosids | Vitales | *Vitis aestivalis* | NC_029454.1 |
| 11 | Ingroup | Malvids | Myrtales | *Eucalyptus deglupta* | NC_022399.1 |
| 12 | Ingroup | Malvids | Myrtales | *Corymbia henryi* | NC_028409.1 |
| 13 | Ingroup | Malvids | Myrtales | *Angophora costata* | NC_022412.1 |
| 14 | Ingroup | Malvids | Brassicales | *Aethionema cordifolium* | NC_009265.1 |
| 15 | Ingroup | Malvids | Brassicales | *Brassica rapa* | NC_040849.1 |
| 16 | Ingroup | Malvids | Brassicales | *Cardamine amara* | NC_036962.1 |
| 17 | Ingroup | Malvids | Huerteales | *Tapiscia sinensis* | NC_036960.1 |
| 18 | Ingroup | Malvids | Malvales | *Gossypium aridum* | NC_033396.1 |
| 19 | Ingroup | Malvids | Malvales | *Daphne kiusiana* | NC_035896.1 |
| 20 | Ingroup | Malvids | Malvales | *Aquilaria sinensis* | NC_029243.1 |
| 21 | Ingroup | Malvids | Sapindales | *Xanthoceras sorbifolium* | NC_037448.1 |
| 22 | Ingroup | Malvids | Sapindales | *Acer buergerianum* | NC_034744.1 |
| 23 | Ingroup | Malvids | Sapindales | *Dipteronia dyeriana* | NC_031899.1 |
| 24 | Ingroup | Malvids | Sapindales | *Citrus aurantiifolia* | NC_024929.1 |
| 25 | Ingroup | Malvids | Sapindales | *Clausena excavata* | NC_032685.1 |
| 26 | Ingroup | Malvids | Sapindales | *Glycosmis mauritiana* | NC_032686.1 |
| 27 | Ingroup | Malvids | Sapindales | *Spondias bahiensis* | NC_030526.1 |
| 28 | Ingroup | Malvids | Sapindales | *Pistacia vera* | NC_034998.1 |
| 29 | Ingroup | Malvids | Sapindales | *Mangifera indica* | NC_035239.1 |
| 30 | Ingroup | Fabids | Oxalidales | *Averrhoa carambola* | NC_033350.1 |
| 31 | Ingroup | Fabids | Malpighiales | *Linum usitatissimum* | NC_036356.1 |
| 32 | Ingroup | Fabids | Malpighiales | *Atuna racemosa* | NC_030546.1 |
| 33 | Ingroup | Fabids | Malpighiales | *Erythroxylum novogranatense* | NC_030601.1 |
| 34 | Ingroup | Fabids | Celastrales | *Euonymus hamiltonianus* | NC_037518.1 |
| 35 | Ingroup | Fabids | Celastrales | *Euonymus japonicus* | NC_028067.1 |
| 36 | Ingroup | Fabids | Celastrales | *Euonymus schensianus* | NC_036019.1 |
| 37 | Ingroup | Fabids | Rosales | *Eriobotrya japonica* | NC_034639.1 |
| 38 | Ingroup | Fabids | Rosales | *Boehmeria spicata* | NC_036989.1 |
| 39 | Ingroup | Fabids | Rosales | *Cannabis sativa* | NC_027223.1 |
| 40 | Ingroup | Fabids | Fagales | *Juglans major* | NC_035966.1 |
| 41 | Ingroup | Fabids | Fagales | *Alnus glutinosa* | NC_039930.1 |
| 42 | Ingroup | Fabids | Fagales | *Corylus chinensis* | NC_032351.1 |
| 43 | Ingroup | Fabids | Cucurbitales | *Gynostemma longipes* | NC_036140.1 |
| 44 | Ingroup | Fabids | Cucurbitales | *Citrullus rehmii* | NC_035975.1 |
| 45 | Ingroup | Fabids | Cucurbitales | *Momordica charantia* | NC_036807.1 |
| 46 | Ingroup | Fabids | Zygophyllales | *Larrea tridentata* | NC_028023.1 |
| 47 | Ingroup | Fabids | Zygophyllales | *Tetraena mongolica* | MK_331720 |
| 48 | Ingroup | Fabids | Zygophyllales | *Zygophyllum fabago* | MK_341052 |
| 49 | Ingroup | Fabids | Zygophyllales | *Zygophyllum xanthoxylon* | MK_296396 |
| 50 | Ingroup | Fabids | Fabales | *Ammopiptanthus nanus* | NC_034743.1 |
| 51 | Ingroup | Fabids | Fabales | *Glycine max* | NC_007942.1 |
| 52 | Ingroup | Malvids | Geraniales | *Viviania marifolia* | NC_023259.1 |
| 53 | Ingroup | Malvids | Geraniales | *Pelargonium x hortorum* | NC_008454.1 |
| 54 | Ingroup | Malvids | Geraniales | *Pelargonium incrassatum* | NC_031200.1 |
| 55 | Ingroup | Malvids | Geraniales | *Pelargonium myrrhifolium* | NC_031201.1 |
| 56 | Ingroup | Malvids | Geraniales | *Erodium chrysanthum* | NC_027065.1 |
| 57 | Ingroup | Malvids | Geraniales | *Erodium gruinum* | NC_025907.1 |
| 58 | Ingroup | Malvids | Geraniales | *Erodium absinthoides* | NC_026847.1 |
| 59 | Ingroup | Malvids | Geraniales | *Geranium incanum* | NC_030045.1 |
| 60 | Ingroup | Malvids | Geraniales | *Geranium palmatum* | NC_014573.1 |
| 61 | Ingroup | Malvids | Geraniales | *Geranium maderense* | NC_029999.1 |
| 62 | Ingroup | Malvids | Geraniales | *Erodium texanum* | NC_014569.1 |
| 63 | Ingroup | Malvids | Geraniales | *Erodium crassifolium* | NC_025906.1 |
| 64 | Ingroup | Malvids | Geraniales | *Erodium manescavi* | NC_030720.1 |
| 65 | Ingroup | Malvids | Geraniales | *Erodium trifolium* | NC_024635.1 |
| 66 | Ingroup | Malvids | Geraniales | *Erodium reichardii* | NC_030721.1 |
| 67 | Ingroup | Malvids | Geraniales | *Erodium carvifolium* | NC_015083.1 |
| 68 | Ingroup | Malvids | Geraniales | *Erodium rupestre* | NC_030719.1 |
| 69 | Outgroup | Basal angiosperms | Amborellales | *Amborella trichopoda* | NC_005086.1 |
