# Supplementary material for: The complete chloroplast genome sequencing analysis revealed an unusual IRs reduction in three species of subfamily Zygophylloideae
Source: PLoS One. 2022 Feb 2;17(2):e0263253. doi: 10.1371/journal.pone.0263253 (PMC8809528; doi:10.1371/journal.pone.0263253)
Supplement: S3 Table — (DOCX) [file pone.0263253.s003.docx]

**S3 Table. Reported chloroplast genomes with size smaller than 130 kb and tobacco chloroplast genome.**

| **Species** | **Family** | **Accession** | **Size(bp)** | **LSC (bp)** | **SSC (bp)** | **IR (bp)** | **Number of genes** |
| --- | --- | --- | --- | --- | --- | --- | --- |
| *Welwitschia mirabilis* | Welwitschiaceae | NC_010654.1 | 119,726 | 68,556 | 11,156 | 20,007 | 101 |
| *Gnetum ula* | Gnetaceae | NC_028734.1 | 113, 249 | 64,914 | 8,791 | 19,772 | 116 |
| *Cuscuta chinensis* | Convolvulaceae | MH780079.1 | 86,927 | 50572 | 7,121 | 14,617 | 85 |
| *Cuscuta japonica* | Convolvulaceae | MH780080.1 | 121,037 | 79,517 | 8,412 | 16,554 | 96 |
| *Astragalus membranaceus* | Fabaceae | KU_666554.1 | 123,582 | 80,986 | 13,772 | 28,822 | 110 |
| *Taxillus chinensis* | Loranthaceae | NC_036306.1 | 121,363 | 70,357 | 6,082 | 22,462 | 106 |
| *Taxillus sutchuenensis* | Loranthaceae | NC_036307.1 | 122,562 | 70,630 | 6,102 | 22,915 | 106 |
| *Epifagus virginiana* | Orobanchaceae | NC_001568.1 | 70,028 | 19,799 | 4,759 | 22,735 | 53 |
| *Nicotiana tabacum* | Solanaceae | NC_001879.2 | 155,943 | 86,686 | 18,571 | 25,341 | 146 |
